# Alternative Splicing Variation: Accessing and Exploiting in Crop Improvement Programs

**DOI:** 10.3390/ijms242015205

**Published:** 2023-10-15

**Authors:** Sangam L. Dwivedi, Luis Felipe Quiroz, Anireddy S. N. Reddy, Charles Spillane, Rodomiro Ortiz

**Affiliations:** 1 Independent Researcher, Hyderabad 500016, India; sangam375@gmail.com; 2 Agriculture and Bioeconomy Research Centre, Ryan Institute, University of Galway, University Road, H91 REW4 Galway, Ireland; luis.quiroz@universityofgalway.ie (L.F.Q.); charles.spillane@nuigalway.ie (C.S.); 3 Department of Biology and Program in Cell and Molecular Biology, Colorado State University, Fort Collins, CO 80523, USA; anireddy.reddy@colostate.edu; 4 Department of Plant Breeding, Swedish University of Agricultural Sciences, 23053 Alnarp, SE, Sweden

**Keywords:** alternative splicing, biological rhythms, domestication and polyploidization, gene mining, heterosis, nutrient homeostasis, plant phenology and architecture, symbiosis, transcriptome and proteome diversity

## Abstract

Alternative splicing (AS) is a gene regulatory mechanism modulating gene expression in multiple ways. AS is prevalent in all eukaryotes including plants. AS generates two or more mRNAs from the precursor mRNA (pre-mRNA) to regulate transcriptome complexity and proteome diversity. Advances in next-generation sequencing, omics technology, bioinformatics tools, and computational methods provide new opportunities to quantify and visualize AS-based quantitative trait variation associated with plant growth, development, reproduction, and stress tolerance. Domestication, polyploidization, and environmental perturbation may evolve novel splicing variants associated with agronomically beneficial traits. To date, pre-mRNAs from many genes are spliced into multiple transcripts that cause phenotypic variation for complex traits, both in model plant *Arabidopsis* and field crops. Cataloguing and exploiting such variation may provide new paths to enhance climate resilience, resource-use efficiency, productivity, and nutritional quality of staple food crops. This review provides insights into AS variation alongside a gene expression analysis to select for novel phenotypic diversity for use in breeding programs. AS contributes to heterosis, enhances plant symbiosis (mycorrhiza and rhizobium), and provides a mechanistic link between the core clock genes and diverse environmental clues.

## 1. Alternative Splicing Isoforms as Source of Transcriptome and Proteome Diversity Contribute to Phenotypic Variation

Transcript expression and alternative splicing (AS) are two key pre-translational processes, which can generate phenotypic variation for all organisms [1,2]. While transcript expression levels are largely dependent on the interplay between promoter and enhancer activities regulating transcription rates and the rate of RNA degradation (or decay), AS can alter the transcript structure leading to modifications of the encoded protein structure to generate different protein isoforms or protein variants [3]. Alternative splicing can also alter the two-dimensional and three-dimensional structure of RNA transcripts, with possibilities for altered functionality at the non-coding transcript level.

Regardless of the close spatio-temporality shared between transcript expression and AS, it has been considered that these two processes are independent of each other [4,5]. However, the regulatory relationship connecting these processes remains unclear, and emerging evidence in both plants and animals underscores the significant influence of transcription on splicing regulation [6,7,8,9]. Because AS can produce new protein variants, it has been suggested that AS is a major source of transcriptome and proteome diversity in eucaryotes, with ultimate impacts on phenotypic variation [10]. Supporting this, a positive correlation has been indicated between the percentage of genes subject to AS and organismal complexity, measured in terms of unique number of cell types [11]. Nonetheless, the role of AS in evolutionary processes, such as speciation and adaptation, remains largely unexplored [8,9,10].

Most transcriptome research has tended to focus on the relative expression levels of mRNA transcripts, both spatially and temporally. This emphasis has arisen due to the relative ease of investigating transcript expression levels with sequencing technologies and bioinformatic tools [12,13,14,15]. Within the canon of transcriptome research, only a sub-set has a focus on AS variation. However, from a functional viewpoint, there is a paucity of investigations that ascribe clear functional effects between the genome-wide extent of AS over time and space and major phenotypic effects. Hence, it is unclear whether AS is a major source of standing genetic variation that in turn generates phenotypic variation. This lack of clarity likely arises from the experimental challenges associated with functionally characterizing the effects of alternative splice isoforms on phenotype [16,17]. Understanding the functional effects of AS isoforms remains a complex task [10,11]. An increasing number of investigations are revealing the growing significance of AS in evolutionary processes, employing advanced techniques such as whole-transcriptome mRNA sequencing [18].

In plants, the domestication process has generated multiple examples of rapid adaptation via AS [19,20,21,22]. One example of this is the *EARLY MATURITY 8* (*EAM8*) gene in barley [19], which is an orthologue of the circadian core component *EARLY FLOWERING 3* (*ELF3*) in *Arabidopsis thaliana*. It has been demonstrated that a mutated version of *EAM8* (*eam8.I*), carrying an A to G transition in position 3257 at intron 3, which leads to an AS event with intron retention and a putative truncated protein, is responsible for the early flowering of a barley landrace from the Tibetan plateau, which is a short-season adaptation to high latitudes [19]. Another example of AS is in the domestication of sunflowers, where the domestication process (approximately 5000 years ago) was associated with a large frequency of alternative transcript isoforms generated by AS. In the AS analysis of sunflower domestication, both new combinations of ancestral spliced genes were found and also novel isoforms [20,21]. These examples suggest that AS can be an important component in evolution and domestication contributing to phenotypic variation within and between natural and domesticated populations. 

In addition to phenotypic variation, phenotypic plasticity is also a key force in evolution and adaptation [23,24]. While the role of transcript expression is well understood, little is known regarding the potential of AS to generate phenotypic plasticity [10,25]. In plants, the association of environment-triggered AS with environmental stress responses suggests that AS could act as a “molecular thermometer” [26]. However, the role and underlying mechanisms by which AS can produce plastic phenotypes in novel ecological or environmental contexts are largely unexplored. 

## 2. Bioinformatic Tools, Software, and Computational Methods to Quantify and Visualize Splicing Variants

Transcriptome-wide analyses of AS in tissues and plants subjected to various biotic and abiotic stresses as well as in different cultivars have been performed using high-throughput next-generation sequencing technologies, such as Illumina RNA-seq (RNA-seq) [27,28,29], Pacific Biosciences single-molecule real-time (SMRT) long-read Isoform sequencing (PacBio Iso-seq) [30,31], and Oxford Nanopore direct RNA sequencing (dRNA-seq), also called native RNA sequencing [29,32,33,34,35]. All these technologies involve sequencing of fragments of total cellular RNA (ribodepleted/poly(A)) or chromatin-associated RNA converted into cDNAs (Illumina short reads), full-length cDNAs (PacBio Iso-seq and Oxford dRNA-seq), or full-length RNAs (Oxford dRNA-seq). Among these, RNA-seq using the Illumina platform has been widely used as it is cheaper and yields more reads. Large-scale Illumina RNA-seq research allowed the prediction of AS events [36,37,38,39]. However, there are limitations with Illumina short reads. The transcript assemblies from short reads are often inaccurate and produce large numbers of mis-assembled transcripts and missing real transcripts [40,41]. Also, research has shown that it is difficult to reconstruct splice isoforms and quantify differential expression of isoforms using short reads [42,43], which is necessary to determine the nature of the encoded protein and in assessing a splice variant’s role [32,43]. To overcome these limitations with short reads, PacBio Iso-seq, which provides long reads, has been used for accurate identification of full-length splice variants and other post-transcriptional regulatory events, such as alternative transcription start sites and alternative polyadenylation sites [30]. As compared to Illumina RNA-seq, PacBio Iso-seq provides more comprehensive insights into different splicing events and isoform diversity and tissue-/condition-specific splicing regulations. Since 2016, Iso-seq has been used to analyze the splice isoforms in several plants, and this has provided a more detailed and in-depth view of numerous novel splice isoforms [30,31,44,45,46,47]. The most recent annotated splice isoforms in AtRTD3 (*Arabidopsis thaliana* Reference Transcript Database3) were assembled with PacBio Iso-seq and Illumina using RNA from many organs/tissues that were subjected to different stresses [31]. The Oxford Nanopore sequencing, which also provides long reads of cDNA or RNAs (dRNA-seq), has been increasingly used in recent years to predict splice isoforms and other post-transcriptional processes including base modifications. Other specialized high-throughput technologies, such as Ribo-seq, are used to assess the translation of splice isoforms [48,49]. Although Iso-seq and dRNA-seq approaches can generate full-length transcript sequences, the major issues are the limited depth in coverage and high error rates, which generate many mis-annotated transcripts [30,31,50,51]. Self- or hybrid-correction methods have been used to overcome the effects of sequencing errors in long reads [30,31,51]. Self-correction uses the raw signal and consensus-based calls to reduce errors, while hybrid correction uses Illumina short reads to correct errors in the long reads. Despite the shortcomings of each of these methods, global research of AS in plants revealed enormous complexity of plant transcriptomes and their regulation at the co-/post-transcriptional levels [29,31,32,52,53,54]. In plants, pre-mRNAs of about 80% of intron-containing genes undergo AS, an essential regulatory mechanism in many developmental and physiological processes that affects thousands of genes [28,31,55,56,57]. For example, research has shown that about 25% of genes that respond to cold stress are regulated by AS [58] and 20 splicing regulators of the SR family produce close to 100 distinct transcripts [59,60,61]. Intron retention is the predominant form of AS in plants, whereas exon skipping is the most prevalent AS event in animals [55,62,63]. However, Braunschweig et al. [64] have shown that IR is highly prevalent in mammals. Research has shown that IR is a regulated process that plays a role in development, stress responses, and disease [64,65,66,67].

Accurate reconstruction of transcript isoforms and quantification of the relative abundance of individual splice isoforms are necessary for a comprehensive analysis of transcriptomes and to decipher the biological functions of individual transcripts. Many computational pipelines have been developed to analyze RNA-seq data to identify AS events, estimate isoform abundance, and differential expression of splice variants across tissues/conditions. Some of the tools/pipelines used for AS analysis are shown in Table 1. These methods use different statistical models, and each has advantages and disadvantages [30,68,69,70]. Depending on the type of reads (short or long reads) and sequencing platform, different computational methods are used. These methods involve the alignment of sequence reads to the reference genome (or reference transcriptome in some cases) and allow detection of specific splicing events (exon skipping, intron retention, alternative 3′ and 5′ splice sites, etc.) and full-length splice isoforms in some cases thereby providing insights into their functional implications. There are also de novo assembly tools, but these methods are highly prone to the assembly of erroneous transcripts [71,72]. More recently, machine learning tools especially deep learning methods are being increasingly used to develop models that can accurately predict splicing/AS patterns of pre-mRNAs and gene expression from genome sequences in humans [73,74,75,76,77,78,79]. These methods are yet to be applied to splicing analysis in plants. The deep learning models determine splicing determinants directly from the nucleotide sequence [73], splice site strength in tissues [74], and impact of genetic variation on RNA splicing [74,75]. These emerging methods offer new ways to predict tissue-/condition-specific AS and the effects of genetic variation in plants on the splicing of protein-coding and protein non-coding RNAs and the biological significance of splicing changes. 

Different genome browsers including Integrative Genomics Viewer (IGV—https://software.broadinstitute.org/software/igv/; accessed on 9 October 2023), Integrated Genome Browser (IGB—https://www.bioviz.org/; accessed on 9 October 2023), or UCSC Genome Browser (https://genome.ucsc.edu/; accessed on 9 October 2023) allow loading of aligned files (BAM files) to visualize sequence depth corresponding to each exon and intron and AS events. The Sashimi plot tool that is part of MISO (mixture of isoforms—https://miso.readthedocs.io/en/fastmiso/; accessed on 9 October 2023) software, which is also available on IGV (https://software.broadinstitute.org/software/igv/Sashimi; accessed on 9 October 2023) takes RNA-seq alignment files (BAM files) and gene annotations as input and provides a comprehensive view of AS patterns. The output plot shows gene structure including exons and introns, splice junctions, AS events, read coverage, and relative abundance of splice isoforms across tissues/conditions. Isoform expression levels and individual splice events, such as the percent “Splice In” of an AS event across samples, can also be visualized using heatmaps [80]. Absolute quantification of splice isoforms in tissues or in response to signals can also be performed using “Quant AS” using a combination of quantitative PCR and digital PCR. 

**Table 1:** Some commonly used tools to analyze RNA-seq data for alternative splicing.

Tool/Pipeline *	Sequencing Platform	Splicing Analysis	URL Address	Reference
**ASpli**	Illumina short reads	Annotated and novel AS events	https://bioconductor.org/packages/release/bioc/html/ASpli.html; accessed on 9 October 2023	[69]
**rMATS**	Illumina short reads; Requires replicates	Differential AS events	https://rnaseq-mats.sourceforge.net/; accessed on 9 October 2023	[81]
**DEXSeq**	Illumina short reads	Differential exon usage	https://bioconductor.org/packages/release/bioc/html/DEXSeq.html; accessed on 9 October 2023	[82]
**MAJIQ**	Illumina short reads	Known and novel local splice variations	https://majiq.biociphers.org/; accessed on 9 October 2023	[83]
**3D RNA-seq**	Illumina short reads	GUI-based pipeline to analyze differential AS and transcript isoforms	https://3drnaseq.hutton.ac.uk/app_direct/3DRNAseq/; accessed on 9 October 2023	[31,70]
**TAPIS**	PacBio Iso-seq	Analysis of AS events and transcript isoforms	https://bitbucket.org/comp_bio/tapis/src/master/; accessed on 9 October 2023	[30]
**SUPPA2**	Illumina short reads	Differential splicing across multiple conditions	https://github.com/comprna/SUPPA; accessed on 9 October 2023	[84]
**TAMA**	PacBio Iso-seq	Transcript isoforms	https://github.com/GenomeRIK/tama; accessed on 9 October 2023	[31,51,85]
**MISO**	Illumina short reads	Differentially spliced exons	https://miso.readthedocs.io/en/fastmiso/; accessed on 9 October 2023	[86]
**SpliceGrapher**	Illumina short reads	Detects patterns of AS	https://splicegrapher.sourceforge.net/; accessed on 9 October 2023	[87]
**iDiffIR**	Illumina short reads	Differential intron retention	https://bitbucket.org/comp_bio/idiffir/src/master/; accessed on 9 October 2023	[88]
**DARTS**	Illumina short reads; Uses a deep learning model and incorporates the expression of RBP.	Differential AS	https://github.com/Xinglab/DARTS; accessed on 9 October 2023	[79]
**SpliceAI**	Illumina short reads; Uses a deep learning model	AS events and splice isoforms	https://github.com/Illumina/SpliceAI; accessed on 9 October 2023	[73]
**Pangolin**	A deep learning model that predicts RNA splicing from DNA sequence	Predicts effects of genetic variants on splicing; tissue-specific splicing	https://github.com/tkzeng/Pangolin; accessed on 9 October 2023	[74]
**SpliceVault** **Web portal**	Uses RNA-seq data	Genetic variant’s effect on splicing	https://kidsneuro.shinyapps.io/splicevault/; accessed on 9 October 2023	[76]

* This list is not comprehensive. Older versions of some of the tools are not listed. Also, some tools for which weblinks are inactive are not included. DARTS, deep-learning augmented RNA-seq analysis of transcript splicing; MAJIQ: Modeling Alternative Junction Inclusion Quantification; MISO, Mixture of Isoforms; RBP, RNA-binding protein; rMATS, Replicate Multivariate Analysis of Transcript Splicing; SUPPA2, Sequencing Unified Pipeline for Proximal Alternative splicing analysis2; TAMA, Transcriptome Annotation by Modular Algorithms.

## 3. Mining Gene Pools for Splicing Isoforms and Diversifying Gene Functions to Obtain Novel Phenotypic Diversity

Alternative splicing allows a gene to encode for various proteins because its exons are put together differently, thus resulting in related but distinct mRNA transcripts. It has been demonstrated that thale cress (*Arabidopsis thaliana*) uses AS disproportionally as a stress response [28,89]. There are other plants showing a cell memory to environmental stress, such as heat [90], which leads to a response to an increase in temperature. Moreover, a synthesized *Brassica* hexaploid had significant AS events [91], thus diversifying its gene expression patterns that could improve its adaptability. Furthermore, Zhang et al. [92] indicated that many genes contributing to quantitative traits are likely to be spliced into multiple transcripts causing their variation. 

The availability of both genome and transcript sequences in plants enables a thorough analysis of AS in various species, including crops [93]. Multi-variate analysis of transcript splicing (MATS) and replicate MATS (rMATS) are robust and flexible statistical software that detect differential AS between two RNA-Seq samples [94] or replicate RNA-Seq data [81], respectively. The synthetic programming of AS patterns, however, remains underexploited for improving crops [95]. Hence, Pramanik et al. [96] suggested CRISPR/Cas9-mediated engineering for modifying AS with the aim of (de)regulating plant development. 

Genome-wide mapping led to the identification of thousands of AS mRNAs isoforms in thale cress [36]. Most of the AS transcripts related to isoforms with premature termination codons, which could shift under abiotic stress. Li et al. [97] did a search of AS affecting reproductive development of young panicles as well as both unfertilized and fertilized florets in rice with the aid of direct RNA sequencing, small RNA sequencing, and degradome sequencing. They found 35,317 AS events, of which in excess of two thirds were novel, and concluded that AS was significantly related to development stages and to complex gene regulation in rice. An RNA-seq survey was able to define AS patterns and to determine that 59.3% of expressed multi-exon genes underwent AS in seedlings, flowers, and young developing fruits of tomato [98]. The use of a single molecule long-read sequencing (Iso-Seq) led to an integrated transcriptome data analysis that facilitated investigating AS in polyploid cotton [99]. This Iso-Seq data analysis was able to identify 15,102 fiber-specific AS events and notice that about 51.4% of homeologous genes produce divergent isoforms in each cotton sub-genome. 

## 4. Molecular Mechanisms Regulating Stress-Dependent Gene Splice Variants

Numerous RNA-seq investigations with plants subjected to various biotic and abiotic stresses have revealed that AS of pre-mRNA is widespread. Furthermore, stresses and developmental cues have a profound impact on the splicing patterns of many genes [28,29,31,44,45,46,47,59,100,101,102,103,104,105,106,107]. Despite the prevalence of AS and its role in stress responses, the regulatory mechanisms of splicing and functions of most splice isoforms are not well understood in plants. Decoding the splicing code in plants would require a comprehensive understanding of the rules that dictate splice site choice and the identification of specific mRNA targets of splicing regulators. A variety of factors including splice site strength and the presence of exonic and intronic splicing enhancers and suppressors affect splice site choice, and RNA structural features [108,109,110] also contribute to AS. Limited research with plants has shown that sequence elements are one of the important determinants of splice site choice [111,112,113,114]. Interestingly, the alternatively spliced genes are over-represented in functional categories related to splicing regulators and stress responses [36,103,115,116]. RNA-binding proteins, such as serine-/arginine-rich (SR) and heterogeneous nuclear ribonucleoproteins (hnRNPs), are some of the key regulators of splicing. Alternative splicing of plant pre-mRNAs encoding SR proteins is dramatically altered in response to various stresses [56,59,117,118,119,120,121,122]. The changes in the levels of these splicing regulators in response to stresses may change the splicing of other pre-mRNAs due to auto- and cross-regulation of splicing [111,123,124,125,126,127]. These investigations suggest that altered ratios of splice variants of splicing regulators in response to stresses may have a role in fine-tuning gene expression at the mRNA and protein levels and the adaptation of plants to stresses [28,128]. Also, many stress-responsive genes are associated with significant splicing quantitative trait loci (sQTLs) in *Arabidopsis thaliana* ecotypes, suggesting a role of AS in plant stress responses [129]. 

There are several hundred RNA-binding proteins (RBPs) in any given plant species, and the precise roles of most of these proteins in co-/post-transcriptional processes are unknown [130]. Many approaches to identifying the roles of RBPs in splice site choice are available, and a comprehensive review of these methods was recently published [29,131], which is why they are not covered in any detail here. In animals, in vitro splicing assays have greatly contributed to our understanding of the roles of spliceosomal and other splicing regulatory proteins in splicing and elucidating steps in spliceosome assembly and spliceosome composition. However, the lack of a robust plant-derived in vitro splicing system in plants has been a major limitation [132]. Hence, other biochemical, cell biological, genetic, and genomic approaches are used to understand splicing regulation in plants [28,29,103,109,133]. The application of new methodologies, such as the identification of targets of RNA-binding proteins using TRIBE (targets of RNA-binding proteins identified by editing) [133,134] and targeted isoform degradation with CRISPR/Cas13 variants [135], may provide insights into targets of hundreds of uncharacterized RNA-binding proteins and elucidation isoform factions. With TRIBE, the targets of an RNA-binding protein are edited irreversibly by de-aminating adenosine to inosine, which is then recognized as guanosine in cDNAs [136] or modified inosines can be identified directly with Nanopore native RNA sequencing [137]. RNA from the RBP-ADAR-expressing plants is sequenced to identify the RNA targets of the RBP by edited events. Expressing specific isoforms in the mutant background or degrading specific isoforms using CRISPR/Cas13 variants (e.g., Cas13d and Cas13x) that specifically bind RNA [135] open a novel and efficient way to study the functions of splice isoforms.

Emerging evidence suggests that stresses/external cues converge on splicing regulators via different signaling pathways. For example, two proteins (Highly ABA-Induced 1 (HAI1), a protein phosphatase 2C and its interacting RNA-binding protein, HIN1(HAI interactor 1, HIN1), an RNA binding protein) involved in drought acclimation interact with the SR family of splicing factors and regulate splicing [107]. Phytochromes, key light receptors and regulators of many aspects of plant growth and development, interact directly with several splicing regulatory proteins and modulate AS of many pre-mRNAs [103,138,139,140]. The light- and drought-regulated alternatively spliced transcripts contain GAA repeats [107,138] that are known to bind splicing regulators (e.g., SCL33, SCL30, and SR45), suggesting that stress-signaling pathways could converge on these splicing regulators [111,113,141]. A mutant of SR45, which encodes a splicing factor, showed altered responses to abiotic and biotic stresses [142,143]. Like abiotic stresses, biotic stresses also change the splicing patterns of many genes. Recent research shows that pathogens effectors modulate host pre-mRNA splicing by binding to splicing regulators, such as serine-/lysine-/arginine-rich proteins, U1-70K, SR30, SR45, and GRP7, and suppress plant immunity [80,144,145,146], suggesting that pathogens have evolved effectors that target host splicing components and subvert plant immunity. It has been shown that many splicing regulators and spliceosomal proteins form speckles (also called biological condensates or membraneless organelles) and stresses alter the dynamics of proteins in these structures and also the size/shape of these structures [133,147,148,149,150,151,152,153,154,155], suggesting that external signals through the re-organization of speckles and their constituent proteins affect pre-mRNA splicing. However, the mechanisms of stress-induced re-organization of speckles in plants are yet to be understood. Also, the phosphorylation status of many spliceosomal proteins and regulatory splicing factors is known to play an important role in pre-mRNA splicing [156] and stresses may alter phosphorylation status and function of splicing regulators.

Until recently, the splicing code has been thought to consist primarily of exonic and intronic sequence motifs that recruit RBPs that either enhance or suppress the selection of nearby splice sites [55,157]. However, in recent years, most pre-mRNA splicing was found to occur co-transcriptionally in both plants and animals [53,54,158], suggesting that chromatin state may affect splice site choice and AS. Emerging research provides evidence in support of multiple regulatory mechanisms at the chromatin level (open vs. closed chromatin, epigenetic modifications including histone modifications and DNA methylation) and the speed of transcription as key regulators that determine the outcome of AS in plants [28,159,160]. A rice mutant (*OsMet1-2*) with impaired DNA methylation altered all types of AS events [159]. Also, a mutant with reduced histone H3 lysine 36-specific methyltransferase in rice showed altered intron retention events [160]. In Arabidopsis and rice, open chromatin was found to be associated with intron retention [161]. Higher speeds of transcription in open chromatin regions provide less time for the spliceosomal machinery to recognize and excise introns co-transcriptionally [162,163]. Alternatively, accessible chromatin regions could be the sites of binding for TFs or other regulatory proteins that recruit splicing factors directly or indirectly through chromatin modifications to affect the outcome of splicing [64,164]. The rate of Pol II elongation during transcription was shown to be involved in light-regulated AS of splicing factors [165,166]. A point mutation in Pol II with increased elongation speed increased splicing, indicating a role for Pol II speed in splicing regulation [166]. Furthermore, an increase in two epigenetic changes (H3K4me3 and H3K9ac) increased the rate of transcription elongation and lowered co-transcriptional splicing efficiency [53]. A double mutant, *rz-1b rz-1c*, of hnRNP-like proteins showed impaired splicing of nascent RNAs, suggesting that these proteins promote splicing at the chromatin level [53]. The direct association of RZ-1C with nascent RNAs further supports its role in co-transcriptional splicing [53]. It has been shown that a shift in temperature alters H3K36me3 methylation and AS [167] and a low temperature changes RNA Pol II elongation kinetics and reduces co-transcriptional splicing [168]. The involvement of chromatin modifiers and a mediator complex in splicing regulation was also reported, and some of these proteins interact with spliceosomal proteins [169,170]. A phosphoprotein phosphatase required for Pol II occupancy was found to promote intron excision [171]. Collectively, these investigations indicate that the epigenetic state of chromatin and the dynamics of transcription modulate AS in plants.

One of the key adaptive changes in response to stresses in plants is the post-transcriptional reprogramming of gene expression [172,173]. The resulting transcript isoforms fine-tune gene expression in profound ways to cope with stresses [90,128,174,175,176]. The research discussed above indicates that stresses/external cues through some yet-to-be-elucidated signaling pathways converge on splicing regulatory proteins and chromatin architecture to modulate AS. An in-depth understanding of splicing code in plants and the roles of splice variants will have applications in fine-tuning gene regulation and developing stress-resilient crops as stresses and developmental cues dramatically alter the levels of splice variants that encode proteins involved in stress responses and plant growth and development [28,29,103].

## 5. Global Expression of AS Isoforms in Model Plant Arabidopsis and among Diverse Crops

### 5.1. Arabidopsis

*Arabidopsis thaliana*, as the main model in plants, has been the subject of intensive investigations to better understand the landscape and functional effects of alternative splice isoforms. Several investigations have demonstrated the widespread extent of AS in Arabidopsis, with initial estimations placing the occurrence of AS events at 11.6% across its genome [116]. However, in recent years and due to the advances in high-throughput sequencing technologies, the estimated rate of intron-containing genes subject to AS has risen to 61–70% in *A. thaliana* [39,89]. Around 40% of the AS events detected represent intron retention, as the predominant type of AS in Arabidopsis [39,89]. Comparisons between AS events involving intron retention vs. non-retention, as well as with constitutive introns, has revealed that the size of the retained introns was significatively smaller than the non-retained ones, with no differences in constitutive introns [39]. Interestingly, it was found that from the total number of AS events that affected protein-encoding genes, 30.3% have little or no effect on the coding sequences (only one amino acid difference or AS in the 5′ and 3′ regions), while the remaining 69.7% of AS events significatively affected the encoded proteins [31]. An additional layer of complexity in the Arabidopsis genome has emerged as cryptic introns that are characterized by the presence of splice sites within annotated coding exons. Approximately 1300 cryptic introns (around 14.1% of all retained introns) have been detected, with nearly half of them undergoing in-frame splicing, hence possessing the ability to excise amino acid stretches from the full-length protein, generating novel protein isoforms [39]. Furthermore, it has been suggested that in Arabidopsis, AS may modulate upstream ORF production in response to environmental stresses by extending 5′ UTR sequences [89]. Interestingly, not only has it been proposed that transcript expression and AS are independent mechanisms in Arabidopsis, but also that transcript expression and AS act in an exclusive manner, in which the genetic structure of transcript expression-regulated and AS-regulated genes exhibit differential genomic architecture [89]. This may suggest that transcript expression and AS are non-redundant and non-overlapping, yet they are complementary mechanisms to generate phenotypic effects.

In a comparison of the global landscape of AS between *A. thaliana* and animals, striking divergences in their regulatory roles have been identified. While animals have harnessed AS as a powerful source of transcriptomic and proteomic diversity, primarily facilitating cellular and tissue specialization, plants have shaped AS into a regulatory mechanism to respond to the ever-changing demands of their sessile lifestyle [89]. For plants, fast and efficient adaptation to shifting environmental conditions and stressors necessitates an AS machinery that can orchestrate in situ responses [89,90]. The divergent evolutionary trajectories of these lineages have led to unique molecular regulatory mechanisms, allowing them to exploit the diverse capabilities of AS to meet their specific developmental and physiological requirements [90]. 

### 5.2. Grain and Fiber Crops

AS is involved in plant response to abiotic stresses and in various aspects of plant growth, development, and reproduction. Genome-wide association analysis (GWAS) is a powerful approach to identify genomic regions and genes associated with complex traits. GWAS has also been found useful in providing genome-wide summary statistics of AS variants and in genome-wide association analysis of AS variants associated with complex traits. Genomic regions associated with gene expression are commonly referred to as quantitative trait loci (QTL), whereas those regulated by AS variants are referred to as splicing quantitative trait loci (sQTL). An analysis of population-level transcriptome data and GWAS of splicing QTL in developing maize kernels from 368 maize inbred lines unfolded 19,554 unique sQTL for 6570 genes, with distinct protein functions. Natural variation in AS and overall mRNA levels were independently regulated with different *cis*-sequences used preferentially. Two hundred and fourteen putative *trans*-acting splicing regulators, including *ZmGRP1*, controlled the largest *trans*-cluster, and the knockout of *ZmGRP1* modified splicing of several downstream genes. There were 739 sQTL that colocalized with known trait QTL, indicating the significance of AS in diversifying gene function to regulate phenotypic variation [22]. An earlier study involving teosinte (wild ancestor) and maize transcriptomes reported 13,593 highly conserved genes, including 12,030 multi-exonic genes. The two species were no different in number of AS events. Over 60% of the AS in both species were of intron retention (IR) and alternative acceptor (AA) types. The average number of unique AS events per alternatively spliced gene was higher in maize (4.12) than in teosinte (2.26). Ninety-four genes in maize generated 98 IR types with transposable element (TE) sequences, far more than 9 IR with TEs in teosinte. TE insertion is probably an important mechanism for IR-type AS in maize. The AS levels of 3864 genes were significantly different between maize and teosinte, of which 151 AS level-altered genes involved in transcriptional regulation and in stress responses were located in the regions that were targets of selection during maize genetic improvement [177].

GWAS unfolded 35,317 AS events at the early reproductive stage in rice, of which ~67% were of novel AS isoforms, and the intron retention (IR) sub-type was the most abundant [97]. Over 11,000 novel splice isoforms, alternative polyadenylation (APA) of ~11,000 expressed genes and more than 2100 novel genes were reported in sorghum [30,178], whereas 15,102 fiber-specific AS were reported in cotton [99]. To date, a large number of AS events associated with various development stages and molecular functions have been reported in soybean—294,164 AS events across multiple experiments [177]; 1278 AS events associated with nitrate stress in root hairs [179]; and 154,469 AS events in 23,764 genes across development stages [180]. The intron retention form of AS events was predominant in most of the research reported here followed by alternative acceptor sites, alternative donor sites, and exon skipping.

Polyploidization, an evolutionary force, promotes diversity and evolution of new species. A GWAS analysis of synthesized hexaploidy *Brassica* (2*n* = 54) and its parents unfolded 7913, 14,447, and 13,205 AS genes that produced 27,540, 70,179, and 60,804 AS isoforms in *Brassica rapa* (turnip, 2*n* = 20), *B. carinata* (Ethiopian mustard, amphidiploid, *2n =* 34), and *Brassica* hexaploidy, respectively. Hexaploid *Brassica* has 920 new genes. The number of differentially spliced genes between hexaploidy *Brassica* and its parents was 56. Hexaploid *Brassica* and its parents had diverse AS patterns of genes, including the gain and loss of AS isoforms [91]. 

Maize was domesticated in the tropics but is widely grown in temperate environments. How did variation in gene expression, as measured by changes in transcriptomes, enable maize to adapt in temperate environments? A genome-wide association study involving eGWAS and sGWAS based on 572 unique RNA-seq datasets from the roots of 340 maize lines identified 19,602 eQTL associated with the expression of 11,444 genes and 49,897 sQTL for 7614 genes. Genes containing both *cis*-eQTL and *cis*-sQTL in LD disproportionately encoded TFs associated with one or more stresses. Further, gene expression data listed transcriptional regulatory networks associated with gene expression, cell propagation, and phase transition powered tropical maize adaption in temperate environments [181].

### 5.3. Vegetable Crops

A systematic GWAS analysis of AS events in potato plants revealed 226,769 AS events, of which 49% were classified as basic and 51% as complex events, generated from 24,650 genes. The basic events include 19.2% alternative acceptor sites (AAS), 12.1% exon skipping (ES), 8.2% alternative donor site (ADS), and 9.5% intron retention (IR) types. A comparative analysis detected 2929 AS genes conserved among maize, potato, soybean, and tomato plants [182]. The AS landscape in tomatoes consists of 369,911 AS events, identified from 34,419 genomic loci involving 161,913 transcripts. IR-type AS events were predominant (18.9%) followed by AAS (12.9%), ADS (7.3%), and ES (6.0%) within the basic events. The complex AS accounted for 54.9% of total AS events. Sixty-five percent of 35,768 annotated protein-coding genes had pre-mRNAs generating AS isoform transcripts [183]. Thus, it is a useful genomic resource for functional characterization of genes in potato and tomato biology.

## 6. Genomic Regions Regulating Splicing of Quantitative Trait Loci (sQTLs)

### 6.1. Novel Splicing Variants Impacting Flowering and Plant Architecture

Evolutionary transition from wild species to crops or polyploidization may evolve novel splicing variants and may contribute to adaptation and population divergence. Hexaploid wheat is an ideal model for studying variation in the AS landscape in response to domestication and polyploidization. Transcriptome sequencing of roots and leaves of wheat species differing in ploidy levels unfolded ~22% of the genes exhibiting AS events. However, AS events decreased after domestication and polyploidization. The decrease in AS events is consistent with the functional sharing model that proposes complementarity between the two (AS-duplicated genes) in regulating transcriptome plasticity in polyploid crops. Sub-genomes exhibited biased AS response to polyploidization, with ~87% of homeologs showing AS partitioning in hexaploid wheat, and substitution of the D-sub-genome modified ~43% of AS patterns of the A- and B-sub-genomes. Thus, AS variation occurs extensively after polyploidization and domestication in wheat [184]. 

The regulation of AS in polyploid wheat (tetraploid and hexaploid) and its ancestral diploid grass species unfolded diversity in AS events not only between the endosperm and pericarp and embryo over-development, but also between sub-genomes. The triads of homoeologous chromosomes revealed evolutionary divergence between gene- and transcript-level regulation of embryogenesis. The novel transcript isoforms in young genes were at a more rapid rate than ancient genes, providing a greater understanding of the evolution of regulatory features of AS during embryogenesis and grain development in wheat [185].

The substantial splicing divergence and predominance of divergent splicing transcripts for seed traits between wild and cultivated sunflowers suggest that domestication and selection for seed development affected the evolution of splicing variants in sunflowers. While *Helianthus annuus* (wild species) contributed to most of the differential splicing patterns, other *Helianthus* species also contributed to domestication-associated splicing patterns in sunflowers [21]. Significantly more AS isoforms were reported among wild accessions than domesticated sorghum accessions [186].

A multi-silique trait (zws-ms) was discovered in the rapeseed. Such a line forms three independent siliques instead of a commonly observed single silique, with temperature being the most critical factor likely to switch on/off the formation of multi-silique [187]. The pattern of transcriptome variation between zws-ms and its NIL (zws-217), which produces normal siliques (i.e., single silique) grown under optimal conditions, unfolded in a colder environment 11 differentially expressed alternative splicing (DAS) genes, of which 4 were up-regulated and 7 were downregulated in a multi-silique line. Five such genes were associated with the multi-silique trait [188], and two thermos-morphogenesis genes switched off genes controlling the multi-silique trait in cold environments [189].

Do AS events affect flowering and plant architecture in wheat? The two splicing variants of *TaNAK1* (*TaNAK1.1* and *TaNAK1.2*) show distinct expression patterns during wheat growth and development, while such an effect has not been observed for *TaNAK1.3*. *TaNAK1* is mainly expressed in developing grains, while *TaNAK1.2* is expressed in leaf and flag leaf. Transgenic *Arabidopsis* over-expressing *TaNAK1.1* and *TaNAK1.2* showed opposite effects (i.e., *TaNAK1.2* positively regulates transition from vegetative to reproductive growth, plant height, branching, seed size, and seed yield, while *TaNAK1* negatively regulates these traits) on flowering and plant architecture, resulting in varying seed yield [190]. 

### 6.2. Seed Yield and Quality

Spikelet architecture, seed size, and weight are the major determinants of yield in cereal crops. Five splicing variants in *TaGS3* and *TaGS3.1* to *TaGS3.5* showed expression divergence during polyploidization and differential functions to regulate seed size and weight in wheat. *TaGS3.1* over-expression significantly reduces seed weight and length by 5.89% and 5.04%, respectively. *TaGS3.2*-*3.4* over-expression relative to wild type (WT) had no significant effect on grain size. *TaGS3.5* over-expression significantly increases seed weight and length by 5.7% and 4.3%, respectively [191]. 

Multiple signaling pathways at transcriptional and post-translational levels control GS3, seed size QTL in rice. The dominant AS isoforms of GS3, GS3.1, and GS3.2 account for about 50% and 40% of total transcripts. GS3.1 over-expression decreases seed size, whereas GS3.2 has no significant effect on seed size. GS3.2 interacts with RGB1 to disrupt GS3.1 activity, thereby implying AS of GS3 decreases the amount of GS3.1 and GS3.2 disrupts the GS3.1 signaling to inhibit the negative effects of GS3.1 to fine-tune grain size in rice [192].

Poor seed filling of inferior spikelets is one of the major limitations in raising rice production. Post-anthesis moderate soil drying promotes starch synthesis and seed filling in inferior spikelets. An assessment of AS events at the grain-filling stage in inferior spikelets under control (irrigated, C) and moderate drought (MD) stress unfolded 16,089 AS events, of which 1840 involving 1392 genes occurred differentially between C and MD treatments, many of which function on spliceosome, starch, and sucrose metabolism, providing new insights into the role of AS to promote seed filling in inferior spikelets under MD in rice [193].

Maintaining yield and quality under low nitrogen conditions is a significant production constraint in cereal. *OsGS1;1* enhances nitrogen use efficiency (NUE). SNP polymorphisms in the *OsGS1;1* region led to the discovery of AS that generated two functional transcripts, *OsGS1;1a* and *OsGS1;1b*. Germplasm containing the *OsGS1;1b* haplotype had improved NUE, positively affected seed development, and reduced amylose content, providing a new avenue to raise yield and nutritional quality of rice under low N conditions [194]. *OsLG3b* regulates grain length in tropical Japonica rice. *OsLG3b* expression is higher during the panicle and seed development stages. SNP polymorphism in the *OsLG3b* region discovered AS that was found to be associated with grain length and was extensively used in breeding to enhance the productivity of tropical japonica rice [195]. 

Yellow seed coat color in rapeseed is associated with higher oil content and a higher quality of meal. A comparison of yellow- and black-seeded rapeseed lines at five developmental stages revealed highly similar AS events in the different samples, with the intron retention type being the predominant form of AS patterns. The early and middle stages of seed development were most-affected by AS variants. Twenty-three co-expression modules composed of differentially spliced genes were detected, of which the function of two modules was highly associated with seed coat color. Both the modules in-housed differentially alternative splicing (DAS) candidate genes related to the flavonoid pathway (*TT8*, *TT5*, *TT12*, *AHA10*, *CHI*, *BAN*, and *DFR*), which could be exploited to develop stable, yellow-seeded rapeseed [196]. A splicing error in the phytic acid synthase gene *inositol-1,3,4 triphosphate 5/6-kinase 3* (*GmITPK3*) caused a low seed phytate phenotype in soybean [197]. Low seed phytate crops improve the bioavailability of micronutrients. Food rich in flavonoids promotes human health and minimizes the risk of old age diseases [198]. *BnaPAP2.A7* regulates anthocyanin biosynthesis, with AS (three splicing isoforms) as the main mechanism for the modulation of anthocyanin biosynthesis in rapeseed leaves [199].

### 6.3. Mineral Nutrient Homeostasis

Very limited knowledge exists about the role of AS in maintaining mineral nutrient homeostasis in plants. Using root transcriptome sequencing of rice grown in the presence or absence of minerals (Fe, Zn, Cu, Mn, and P), Dong et al. [106] noted 13,291 alternatively spliced genes, with a small overlap between differentially expressed genes (DEGs) and DAS genes. Nutrient-specific AS genes represented ~53.3% of multi-exon genes in the rice genome. A group of splicing factors known as serine-/arginine-rich (SR) proteins regulate AS mechanisms. The characterization of mutants in gene-encoding Ser/Arg (SR) proteins in rice unfolded several SR proteins as critical regulators of Zn, Mn, and P nutrition, with highly specific AS targets for each nutrient. For example, three SR protein-encoding genes regulate P uptake and re-mobilization between the leaves and shoots of rice [106]. Clearly, this is an under-explored area of research and must be further investigated to unfold the molecular basis of mineral nutrient homeostasis (DEGs, DAS genes, and interaction between DEG and DAS) in plants.

### 6.4. Abiotic Stress Adaptation

Alternative splicing variants increase proteome diversity. Heat shock transcription factor (Hsf) under stress may form different transcripts by AS. A novel splice variant *TaHsfA2-7-AS*, induced by high temperature, regulates thermotolerance in wheat, and its over-expression in *Arabidopsis* enhances tolerance to heat stress [200]. Plant serine-/arginine-rich (SR) proteins contribute to abiotic stress adaptation by regulating AS of key genes. Over-expression of *BrSR45a* in *Arabidopsis* regulates the drought stress response via the AS of target genes in a concentration-dependent manner [201]. Over-expression of AS-related protein from cassava, MeSCL30 in *Arabidopsis*, enhances tolerance to drought via maintaining ROS homeostasis and increasing the expression of drought-responsive genes [202]. 

The interplay of AS under stress and across development stages (ear, tassel, and leaf) of a public inbred line B73 under well-watered and drought stress conditions unlocked over 48,000 novel AS isoforms, often with stage- or condition-specific expression. Stress induces large developmental splicing changes in leaves and ears but only a few in tassels. Most of the developmental stage-specific splicing changes affected by stress are tissue-dependent, whereas stage-independent changes frequently overlap between leaves and ears. This suggests that AS is strongly associated with tissue type, developmental stage, and stress condition [203]. Low-temperature stress reduces seed germination, which in turn results in low plant populations and reduces grain yield in maize. A genome-wide analysis of AS during cold stress unlocked approximately 2.05–2.09 AS events per gene on each chromosome, of which seven exclusively expressed AS in cold-tolerant maize inbred lines. Functional validation of the AS gene through mutation reveals that ZmWRKY48 is associated with low-temperature resistance in maize [204].

SR-rich splicing factors play a key role in pre-mRNA splicing to regulate plant growth and development under stress conditions. Butt et al. [205] investigated the role of the plant-specific SR protein RS33 in regulating pre-mRNA splicing and abiotic stress responses in rice. Loss-of-function mutant *rs33* showed increased sensitivity to salt and low-temperature stresses. They identified multiple splice isoforms of stress-responsive genes whose AS is regulated by RS33, and the expression of RS33-regulated genes was more under cold than salt stress, indicating plant-specific splicing factor RS33 is crucial in response to abiotic stresses [205]. Multiple abiotic stresses also triggered extensive but different expressional regulations on sweet potato *SR* genes. Heat stress caused substantial disturbances in both gene transcription and pre-mRNA AS. Tissue- and species-specific AS regulations in response to stresses were noted in sweet potato, unlike *Arabidopsis* and rice [206].

Multiple abiotic stress induces several DAS genes. Three hundred and fifty-seven DAS genes and their splicing isoforms of candidate genes (*RBP45C*, *LHY*, *MYB59*, *SCL30A*, *RS40*, *MAJ23.10*, and *DWF4*) were induced in rapeseed [207]. In soybean roots, between 385 and 1429 AS events were differentially spliced under varying water deficits and recovery after severe drought stress [208]. In rice, 764 genotype-specific splicing (GSS) events were identified in salt stress conditions, of which six events in five genes were significantly associated with the shoot’s Na^+^ content. *OsNUC1* and *OsRAD23* emerged as candidate genes with splice variants exhibiting significant divergence between the variants for shoot growth under salt stress conditions [209]. In response to drought, heat, and combined (drought and heat) stress in wheat, 200, 3576, and 4056 genes exhibited significant AS pattern changes. The combined stress induced specific AS compared to individual stress, while the B sub-genome exhibited more AS events than on the A and D genomes [210]. The splicing isoforms of candidate genes could be a valuable resource for enhancing abiotic stress adaptation in plants.

The productivity and quality of vegetable crops is impaired in sub-optimal environments. Identifying and exploiting variations in AS events is a powerful approach to cope with these stresses by producing multiple mRNA splicing variants with different sub-cellular localizations, translational efficiency, and coding sequences in vegetable crops [211]. A comparative assessment of AS in tomato plants subjected to varying levels of moisture stress unfolded 464, 512, and 506 AS events under optimal (irrigated), mild, and severe drought stress conditions, respectively. The stage-dependent changes in AS genes may participate in plant tolerance to drought stress [212]. An assessment of differentially expressed genes (DEGs) and differentially alternatively splicing (DAS) events in stress-tolerant (water deficit, low nitrate, or a combination of both) (T270) and intolerant (T250) tomato accessions reveals that transcriptome changes caused by combined stress yielded a low number of DEGs in the tolerant compared to intolerant genotypes. DAS events in combined stress greatly affect the splicing landscape in both genotypes; i.e., stress- and growth-related genes as well as transcription and splicing factors are differentially spliced in the roots and leaves of the two genotypes. This clearly shows that transcriptional and post-transcriptional mechanisms regulate tomato adaptation to growth under drought and low N stress [213]. 

## 7. Alternatively Spliced Variants Contribute to Hybrid Vigor

Heterosis, or the superior performance of F_1_ hybrids vis-à-vis their parental lines, has been widely applied for breeding output to raise the productivity of crops, especially in cross-pollinated grain crops (e.g., maize, pearl millet, and pigeonpea) and in a few self-pollinated crops (e.g., rice, tomato and other vegetables). Differential gene and protein expression between hybrids and their parents regulate hybrid vigor. A genome-wide assessment of AS variants between hybrids and their parents may provide additional means to exploit heterosis in plants. Profiling AS landscape data from immature ears of the maize hybrid ZD808 and its parents (NG5 and CL11) unfolded substantial differential AS events in the hybrid vis-à-vis its parents, which were classified into parental-dominant and novel DAS patterns. NG5-dominant events prevalent in the hybrid accounted for 42% of DAS events and were mainly involved in regulating gene expression associated with carbon/nitrogen metabolism and cell division processes. *Cis*-regulation was the predominant contributor to AS variation and was involved in biological processes associated with immature ear development in maize [214].

In sorghum, the developing embryo and endosperm show significant and multi-faceted differences in gene expression and AS that may potentially correlate with hybrid vigor. An analysis of genome-wide gene expression between developing embryo and endosperm as well as between F_1_ hybrids and their parental lines in sorghum uncovered substantial differences in both gene expression and AS events between embryo and endosperm, which were consistent with their biological roles in the two tissues. The hybrids relative to their parents showed substantial and multi-faceted differences in gene expression and AS events, which were distinct and tissue-specific, and may provide transcriptome resources to further elucidate seed yield heterosis in sorghum [215].

A recent study on a sunflower hybrid under control (irrigated) and drought stress conditions revealed that the ‘absence’ alleles at presence/absence variants (PAVs) were disproportionately associated with reduced values of heterosis-related traits, but not those of non-heterotic traits. The expression of gene PAVs differentiating the parental lines was complemented in hybrids, thereby supporting the dominance model of hybrid vigor and yield stability across environments. The consistent expression of many of the PAVs in control and drought stress conditions possibly contributed to heterosis under drought stress. A further comparison of DEGs between hybrid and parental lines revealed that parents responded similarly to drought stress by up-regulating stress response and down-regulating metabolic process genes, while these responses were further strengthened in the hybrid. An inverse relationship between AS changes and expression changes in DEGs implies that AS acts to reinforce expression responses [216]. 

## 8. Establishing a Platform for Cataloguing, Curating, and Retrieving Alternative Splicing Isoforms and Gene Expression Quantification Database across Tissues, Development, and Stress Conditions

One of the major challenges in researching AS and transcript expression is the fragmented nature of data; AS isoforms and transcript expression data are scattered across many investigations and datasets, often lacking standardized annotations and metadata [217,217]. Such fragmentation hinders efficient data retrieval, comparison, and interpretation. Furthermore, inconsistencies in data formats and quality pose additional obstacles for researchers [217,218]. To address these challenges, the development of unified platforms for cataloguing, curating, and retrieving AS isoforms and GE quantification data is important. In this context, multiple attempts to generate a unified platform for GE and AS have been published to the date [217,218,219,220,221,222]. The aim of such platforms is to provide researchers with a centralized resource for accessing comprehensive and high-quality data across different biological contexts and investigations. 

The accessibility of such platforms requires a focus on user-friendly interfaces, powerful search functionalities, and intuitive data visualization tools. This can allow researchers to better explore and analyze complex AS patterns and transcript expression dynamics. Advanced algorithms and computational tools are being implemented to enable a more comprehensive data analysis, allowing researchers to uncover novel insights into AS and transcript expression [217,218,219,220,221,222]. For instance, the PlantExp platform integrates plant transcript expression and AS profiles from 131,423 uniformly processed publicly available RNA-seq samples that belong to 85 plant species across 24 plant orders [219]. This platform not only allows researchers to investigate and navigate across AS and transcript expression profiles, but also allows a differential and specific expression analysis, an analysis of co-expression networks, a cross-species expression conservation analysis, and an easy visualization of data [219]. 

Such platforms not only facilitate data-driven research, but also promote collaboration between scientists working on AS and transcript expression. By integrating fragmented data, ensuring data quality and accessibility, and providing powerful analysis tools, such platforms empower researchers to explore the intricate relationship between AS and transcript expression. In addition, scientists can flexibly customize sample groups to re-analyze publicly available RNA-seq datasets and obtain new insights [217,218,219,220,221,222]. 

## 9. Alternative Spliced Circadian Clock Genes in Response to Abiotic Stress

Circadian clock genes are a key point of regulation for adaptation to new environments and abiotic stress conditions. Alternative splicing plays a crucial role in the regulation of many core clock genes in plants and represents an important mechanistic link between the core of the circadian clock and diverse environmental inputs [223,224,225,226,227]. One example is partially redundant MYB-related transcription factors CIRCADIAN CLOCK ASSOCIATED 1 (CCA1) and LATE ELONGATED HYPOCOTYL (LHY) [228]. Under cold conditions, a non-functional spliced variant of LHY, which has a premature stop codon, is accumulated [223]. In contrast, the spliced variant of *CCA1β*, which lacks the MYB-like DNA-binding domain by retaining the fourth intron, is inhibited at low temperatures. The result is that *CCA1β* interferes with the formation of CCA1α (functional full-length CCA1) and LHY hetero- and homo-dimers [229]. The over-expression of *CCA1β* reduces freezing tolerance in *Arabidopsis*, while the over-expression of *CCA1α* increases tolerance to cold conditions [229]. Thus, the opposite regulation of *LHY* and *CCA1* under low temperatures has revealed the role of AS in ensuring the balance of LHY and CCA1 under acclimation to low temperatures [223,229]. Moreover, extensive thermal fluctuations lead to a significant increase in CCA1β isoforms, causing shifts in their daily timing [230]. Likewise, other stresses, such as drought and *Pseudomonas syringae* infection, induce similar effects in the accumulation of CCA1β, thereby suggesting that stress-induced instabilities in the central oscillator might be partially compensated by out-of-phase *CCA1* intron retention transcript oscillations [231].

In addition to *CCA1* and *LHY*, other genes of the clock have been shown to display AS related to cold stress. For example, the spliced version of *TIMING OF CAB EXPRESSION1* (*TOC1β*) is transcriptionally increased at low temperatures, while the *ELF3β* is suppressed under the same conditions [225]. Other abiotic stresses are also involved in differential alternative slicing in plants. For instance, heat stress triggers AS to increase the levels of *CCA1β*, *PSEUDO-RESPONSE REGULATOR7β* (*PRR7β)*, *TOC1β*, and *ELF3β*, while saline conditions do not seem to affect AS of the *CCA1*, *PRR7*, *TOC1*, and *ZEITLUPE* (*ZTL*) genes, but reduce the *ELF3β* variant over the *ELE3α*, revealing a role of AS in the regulation of *ELF3* under salt stress [225]. It has been shown that the splice variants of *TOC1* and *ELF3* undergo degradation via the nonsense-mediated decay (NMD) pathway, while the splice variants of other clock genes exhibit insensitivity to NMD [225]. 

Multiple spliceosome components are involved in AS of core plant clock genes. For example, the conserved methyltransferase PROTEIN ARGININE METHYLTRANSFEREASE 5 (PRMT5), involved in histone methylation, regulates AS of *PRR9* [230,232]. Another key spliceosome component involved in AS of circadian genes is SNW/SKI-INTERACTING PROTEIN (SKIP). It is proposed that SKIP regulates AS of *CCA1*, *LHY*, *PRR7*, *PRR9*, and *TOC1* by modulating the recognition of the 5′ and 3′ splice donor and acceptor sites. Conversely, loss of SKIP causes a long-period phenotype [224]. Likewise, mutants of *SPLICEOSOMAL TIMEKEEPER LOCUS 1* (*STIPL1*), a homolog of a human spliceosome protein, also cause a long-period phenotype. Additionally, in *stipl1* mutants, transcript levels of the spliced variants of *CCA1*, *LHY*, *PRR9*, and *TOC1* are altered [233]. It has also been proposed that CCA1 mRNA intron retention modulation, via functional and nonsense/IR transcript ratios, potentially involves the splicing factor SR45 [231].

Core components of the spliceosomal U6 small nuclear ribonucleoprotein complex, *SM-like* (*LSM*) genes, also regulate circadian rhythms in plants. Mutations in *LSM5* or *LSM4* in *Arabidopsis* extend the circadian period by affecting AS more than constitutive splicing [234]. Another spliceosomal small nuclear ribonucleoprotein assembly factor, GEMIN2, has been suggested to attenuate the effects of temperature on the circadian period by regulation of AS of clock genes, such as *CCA1*, *TOC1*, and *PRR9* [235]. Despite these discoveries, the complete details of the molecular mechanisms involved in AS effects on circadian components remain unknown.

## 10. Alternative Splicing Shapes Plant Symbiosis with Mycorrhiza and Rhizobia

Legumes establish a symbiotic relationship with N-fixing soil bacteria, whereas mycorrhiza establish a symbiotic relationship with both monocots and dicots. *Rhizobium* captures atmospheric N to support plant growth and development, while the bacteria use nutrients from the plants to support their own growth [236]. Mycorrhiza in optimal and stressed environments provide nutrients to host plants to improve biomass yield and quality of edible products under optimal and stressed environments [237]. Recent research as discussed herein states that AS variants contribute to the functioning of symbiosis in plants.

### 10.1. Mycorrhiza Symbiosis

Numerous genes regulate the formation of symbiotic structures and bidirectional nutrient exchange between host plant and mycorrhiza fungi. Tomatoes have emerged as a model plant for arbuscular mycorrhizal symbiosis (AMS). AMS in tomatoes up-regulated 3174 protein-coding genes, 42% of which were AS isoforms. Symbiosis consistently induced 24 genes from the ortho groups in eight phylogenetically distant angiosperms. Seven additional ortho groups were specifically induced by AMS in all surveyed dicot AMS-host plants, whereas these orthos were absent or not induced in monocots and/or non-AMS hosts, indicating a continuously evolving AMS-responsive network in addition to a conserved core regulatory module. A tomato symbiotic transcriptome database (https://efg.nju.edu.cn/TSTD accessed on 10 July 2023) may serve as a resource for deep deciphering of the AMS regulatory network [238].

AS regulates transcriptome and proteome diversity and therefore may influence symbiosis. Transcriptome profiling of pea roots in symbiosis with arbuscular mycorrhiza and control (nonsymbiotic) showed highly similar AS profiles. The intron retention type accounted for 67% of the AS types, as noted among plant species in general. Eight genes with AS events specific for mycorrhizal roots were identified, four of which were annotated as encoding an apoptosis inhibitor protein, a serine/threonine protein kinase, a dehydrodolichyl diphosphate synthase, and a pre-mRNA-splicing factor ATP-dependent RNA helicase DEAH1. The isoforms of these genes were up-regulated in mycorrhizal roots. Two such genes with mycorrhiza-specific AS were related to splicing and were part of the feedback loops involved in fine-tuning gene expression during mycorrhization [239]. 

### 10.2. Rhizobium Symbiosis

The Iso-Seq of soybean root tissues inoculated and uninoculated with *Rhizobium* unfolded 200,681 transcripts and covered 26,183 gene loci. Most of the multi-exon loci produced more than one splicing variant. Seven thousand and seventy-four DAS events had highly diverse splicing patterns (i.e., defense- and transport-related processes) during nodule development. The profiling of genes with differential isoform uses unlocked 2008 multi-isoform loci that underwent stage-specific or simultaneous major isoform switches after inoculation. In addition, 157 of 1563 high-confidence long non-coding RNAs (lncRNAs) were also differentially expressed during nodule development [240]. A study involving soybean transcriptome data unfolded key transcription patterns of nodule development, which included 9669 core genes and 7302 stage-specific genes and uncovered 2323 genes that undergo AS events during the nodule developmental stage in nodules compared to roots. Stage-specific changes during nodulation were also noted in DNA methylation that impacted the expression of 1864 genes. Thus, there exists an association among gene expression, AS, and DNA methylation in shaping transcriptome complexity and proteome specificity in developing nodules [5]. The assessment of AS events in the pea nodules and root tips unraveled AS isoforms of four genes, *PsSIP1*, *PsIGN*, *PsWRKY40*, and *PsPR-10*, with pathogens stress response isoforms more highly enriched in nodules than in root tips [241].

## 11. Applied Aspects of Splice Isoforms in Controlling Agricultural Traits

Alternative splicing produces more than one mRNA from a single pre-RNA molecule in plants, thus increasing transcriptome plasticity and proteome complexity [242] and affecting plant metabolism at different development stages [243]. AS provides therefore means for plants to adapt to changing surrounding environments by regulating their fitness, particularly when they grow under stress [244], e.g., in the response of barley’s clock genes to low temperature [226] or during infection of blast fungus in rice [245]. The recent advances in next-generation sequencing coupled with extensive transcriptomic resources have facilitated the understanding of the role of AS in regulating developmental processes in plants for adapting to stress-prone environments [246]. 

Splice variants affect agronomic characteristics in crops, e.g., floral development in cereals [247], seed shattering and weight in rice [248], grain size and weight in wheat [191,249], plant architecture in soybean [250], and nutritional quality in rice [210,251], soybean [252,253], tomato [254], and wheat [255]. Genome-wide association genetic analysis (GWAS) can further reveal how AS variants diversify gene function and regulate variation in crops, as shown by Chen et al. [22] in maize. They found ca. 20,000 unique splicing quantitative trait loci for 6570 genes affecting protein functions in 366 inbred lines.

## 12. Conclusions

AS of pre-mRNA is widespread and the major source of transcriptome and proteome diversity, which in turn generates phenotypic variation. A variety of computational pipelines including deep learning machine tools methods are now available to analyze RNA-seq data to identify AS events, estimate isoform abundance, and differentiate expression of splice variants across tissues/conditions and development stages. 

Domestication and polyploidization (*Brassica* species and wheat) in addition to environmental perturbation cause varying expressions of AS isoforms in plants. *Arabidopsis* uses AS isoforms as a stress response mechanism to enhance its adaptation to a range of geographically diverse agro-ecologies. To date, many AS quantitative trait loci (sQTL) for a large number of genes with distinct protein functions impacting phenology, plant architecture, biomass yield, or quality, including nutrient homeostasis and stress responses, have been reported in grain (maize, rice, sorghum, and wheat), oil (*Brassica* species and soybean), and fiber (cotton) crops. Many of these sQTL colocalize with known pQTL impacting phenotypic variation. Evidence also suggests that AS variants contribute to the functioning of symbiosis (mycorrhiza and rhizobium) in plants and heterosis in grain and oil crops and provide a mechanistic link between the core of the circadian clock genes and diverse environmental stimuli.

Though significant advances in the genome-wide expression of AS variants have been made in various crops, applying such advances poses a significant challenge in crop improvement programs, which include but are not limited to (i) a significant bottleneck to establishing cost-effective high-throughput assays to identify AS variants in early breeding generations; (ii) differentiating and quantifying the impact of sQTL from pQTL for genes impacting phenotypic variation; (iii) accurate reconstruction of transcript isoforms and quantification of relative abundance of individual isoforms in deciphering the biological functions of individual transcripts; (iv) identifying common genetic tags (e.g., SNPs, InDels, and structural variation) linked with AS variants and gene expression; and (v) possible adverse effect of combining AS variants with trait gene(s) on phenotypic variation. Until such logistical issues are resolved, the exploitation of AS variants in crop improvement programs will be limited to the discovery and functional characterization of AS variants across tissues or conditions and development stages in plants.
